# Changes in Human Parechovirus Profiles in Hospitalized Children with Acute Gastroenteritis after a Three-Year Interval in Lanzhou, China

**DOI:** 10.1371/journal.pone.0068321

**Published:** 2013-07-03

**Authors:** Ying Guo, Zhaojun Duan, Yuan Qian

**Affiliations:** 1 Graduate School of Peking Union Medical College, Beijing, China; 2 Laboratory of Virology, Capital Institute of Pediatrics, Beijing, China; 3 National Institute for Viral Disease Control and Prevention, China Center for Disease Control and Prevention, Beijing, China; Iowa State University, United States of America

## Abstract

The changing profile of infection over time for Human Parechoviruses (HPeVs) is not well known and no detailed study has been reported to date in China. This investigation on HPeV infection in hospitalized children in Lanzhou, China revealed variations in epidemiological characteristics after a three-year interval. To assess the changes that had occurred, epidemiological and clinical characteristics of HPeVs were characterized and compared with previously reported data by our group. A comparable positivity rate (25.3%, 73/289) was revealed after the three-year interval with the majority of the infected children (95.9%, 70/73) being younger than two years of age. While a temporal change in the seasonal distribution was noted in the current study, HPeVs were more frequently detected during July to November compared to September to December in the previous study. Changes in HPeV genotypes patterns, a temporal change in the prevalence of HPeV1, a younger susceptible age to HPeV3 compared with HPeV1 and a tendency of older children to be infected with HPeV4 are in contrast to our previous report. HPeV2, a rarely reported genotype, was identified for the first time in China. In addition, an exclusive trinucleotide (GAT) insertion in the HPeV4 nucleotide sequence was identified. However, the profiles of co-infection with other enteric related viruses were similar to our previous findings. In summary, these data suggest temporal variation in the seasonal distribution of HPeV and changing patterns of HPeV genotypes over time in the study region.

## Introduction

Human Parechoviruses (HPeVs) are single-stranded, positive-sense RNA viruses. The genome is approximately 7,400 nucleotides (nt) in length, encoding three structural and seven non-structural proteins flanked by 5′ and 3′ un-translated regions [1]. Due to their distinctive biological and molecular properties, HPeVs were assigned to the genus *Parechovirus* in the family *Picornaviridae* in the seventh report of the international committee on taxonomy of viruses [2]. To date, 16 genotypes have been identified (http://www.picornastudygroup.com/types/parechovirus/hpev.htm) although the number is expected to increase. HPeVs are very common, with virus particles having been detected and isolated from various clinical samples worldwide and high sero-positivity rates in adults having also been reported [3]–[5]. HPeV1 has always been the most prevalent genotype, while HPeV3 is associated with more serious illness in infants [6], [7]. In addition, HPeV3 is the most common genotype identified in cerebrospinal fluid and displays a peculiarly biennial cycle [6], [8]–[15]. Various clinical manifestations from asymptomatic infection to moderate gastroenteritis or respiratory infection, serious sepsis-like illness [7], [16]–[20], encephalitis [21]–[25], encephalomyelitis [26], transient paralysis [5] or sudden infant death syndrome [27] have been reported in HPeV-infected subjects. However, some investigators believe that HPeV may be incidental and not play a significant role in disease outcome [3], [28]–[31]. Although a ubiquitous virus, HPeV has long been neglected and under-diagnosed in clinical practices. Some recent studies have indicated that additional PCR screening for HPeV has increased the detection of a potential viral cause of specific illnesses [6], [7], [9], [32]. More and more investigators suggest that HPeV detection should be incorporated into routine diagnostic systems, or at least as part of second-line screening assays [15], [33]–[38]. For this reason, several multiplex PCRs for HPeVs identification had been developed [22], [39]–[43].

A previous study by our group provided preliminary data on the prevalence of HPeVs in hospitalized children with acute gastroenteritis in Lanzhou, China [30], while some varied epidemiological characteristics unlike the previous data were found out in a recent investigation on the HPeVs in the same region. The changing epidemiology of other viruses over time has been reported [44]–[47], but since HPeV is still a not yet fully recognized pathogen, report about HPeV in this regard is limited and no related study has been reported to date in China. Therefore, the aim of the present study was to determine changes in the epidemiological and other characteristics of HPeV from our previous study following a three-year interval in order to provide baseline information on temporal variation of HPeV infection and the circulation dynamics of the HPeV genotypes in Lanzhou, China.

## Materials and Methods

### Ethics Statement

All samples involved in the current study were obtained from previously archived samples which had been originally collected for another diarrhea-related research project that had been granted ethics approval by the Review Board of the First Hospital of Lanzhou University (Lanzhou, China). Permitting coded reuse of the samples for future study had been enclosed in the written informed consents. Because all of the samples were coded and anonymously archived and no risk of future identification of the sample was present, the Ethics Committee of the Capital Institute of Pediatrics (Beijing, China) granted a waiver of the ethics approval and new consent requirement.

### Patients and Samples

A total of 289 archived fecal samples, which had been collected from 289 (204 boys, 85 girls) children who were hospitalized at the First Hospital of Lanzhou University (Lanzhou City in the northwest of China) due to acute gastrointestinal disorders from 1 July 2009 to 30 June 2010, were used in the current study. These samples were previously collected for another diarrhea-related research project which had adopted a uniform inclusion and exclusion criteria as described previously [30]. All subjects were younger than five years of age. Fecal specimens were stored anonymously at −70°C without additives.

### RNA Extraction

The stored samples were diluted to 10% with the sample diluent of the IDEIA™ EIA kit (OXIOD, Inc.) followed by vortexing and centrifuging. Then 140 µl of clarified supernatants were collected and used for RNA extraction with the QIAamp Viral RNA Mini Kit (Qiagen, Inc.). Extraction was completed by following the manufacturer’s instruction. The elution volume was 60 µl.

### Screening & Typing PCR

The screening and the typing PCR for HPeVs were performed as described previously [30]. Due to the use of degenerate primers in the typing, an alternative touch-down RT-PCR was used in combination with the previous RT-PCR protocols to improve the genotyping performance when necessary (such as further confirmation of the typing-negative samples, or in the cases of poor typing PCR specificity). The touch-down RT-PCR consisted of an RT reaction at 43°C for 1 h followed by a denaturation step for 2 min at 94°C. This was then followed by 10 cycles of 30 s at 94°C, 30 s at 50°C (decreasing by 1 degree per cycle), and 105 s at 68°C, followed by 30 cycles with an annealing temperature of 40°C, and a final extension at 68°C for 5 min. For the second round of the nested-PCR, the thermal profile consisted of a denaturation step at 94°C for 2 min, then 10 cycles of 18 s at 94°C, 21 s at 55°C (decreasing by 1 degree per cycle), and 90 s at 72°C, followed by 30 cycles with an annealing temperature of 45°C and a final extension at 72°C for 5 min.

### Sequencing and Phylogenetic Analysis

Amplicons of the predicted size (i.e. 300 bp) were recovered from 1.5% (w/v) agarose gels (Invitrogen, Inc.) using the QIAquick Gel Extraction Kit (Qiagen, Inc.), cloned into pGEM-T using the pGEM-T Easy Vector System (Promega, Inc.) and transformed into *E. coli* DH5α for subsequent plasmid purification. The plasmids were then sequenced bi-directionally by a commercial sequencing service (Tianyi Huiyuan Bioscience & Technology Inc., Beijing). The sequences of the HPeV-positive products were aligned using ClustalX 2.1 [48] with those of the reference HPeV genotypes containing the complete VP3/VP1 junction region and available in the GenBank database. Phylogenetic analyses were performed using MEGA version 5 [49]. The evolutionary relationship between sequences was inferred using the Neighbor-Joining method with 1,000 bootstrap repetitions. The evolutionary distances were computed using the p-distance method, all positions containing gaps and missing data were eliminated, and the HPeV genotype was identified according to the generated phylogenetic cluster.

### Co-infections and Clinical Data

IDEIA™ EIA kit (OXIOD, Inc.) for enzyme immunoassay detection of rotavirus was used to screen for co-infections of rotavirus according to the manufacturer’s instructions. A multiplex RT-PCR protocol [50] developed to simultaneously detect norovirus GI and GII, sapovirus and astrovirus, and a PCR [50] for detection of adenovirus were referred to test for the presence of these viruses. These methods applied to the detection of co-infections were consistent with our former study [30]. The clinical data for the children, including age, gender, vital signs, clinical symptoms, laboratory findings, bacterial culture, previous use of antibiotics, length of hospitalization, outcome, etc., were retrieved to assess the association of the HPeV with acute gastroenteritis.

### Statistics

Statistical analyses were performed using the SAS System for Windows (SAS Institute Inc.), version 9.1. Chi-square test (or Yates’ chi-square test, if 1≤*T*<5) was used to evaluate “the gender distribution of HPeVs or individual genotypes”, “the detection rates of rotavirus (astrovirus, calicivirus and adenovirus) in HPeV-positive and negative children”, and “the incidence rates of vomiting or respiratory symptoms between children with HPeV-infection alone and HPeV-infected children with co-infection or children without detected pathogen”. The comparisons of “age distribution patterns”, “the durations of vomiting/diarrhea” and “the daily frequencies (times per day) of vomiting/diarrhea” were analyzed by the Kruskal-Wallis rank sum test because the original data did not meet the requirements for parametric statistics. If the multiple comparisons were applied in the statistical calculations, the significance level would be correspondingly adjusted by Bonferroni correction method and specifically indicated; otherwise, a typical level of 0.05 was set as the threshold of *P*.

### Nucleotide Sequence Accession Numbers

The reference sequences employed in the present study were obtained from GenBank with the following accession numbers (indicated in parentheses): HPeV1 strains Harris (L02971, S45208), A1086-99 (AB112485), BNI-R90/03 (EU024630), SH1 (FJ840477), 7555312 (FM178558), PicoBank/HPeV1/a (FM242866), R2594/1990/HUN (GU125390); HPeV2 strain Williamson (AJ005695); HPeV3 strains A308/99 (AB084913), Can82853-01 (AJ889918); HPeV4 strains Fuk2001-282 (AB433630), T75-4077 (AM235750), K251176-02 (DQ315670), R12644/1999/HUN (GU125397); HPeV5 strains CT86-6760 (AF055846), T92-15 (AM235749); HPeV6 strains NII561-2000 (AB252582), BNI-67 (EU022171), 2005-823 (EU077518), SH6 (FJ888592); HPeV7 strain PAK5045 (EU556224); HPeV8 strain BR/217/2006 (EU716175); Ljungan virus (LV) strain 87-012G (EF202833). The nucleotide sequences generated in this study have been deposited in GenBank under accession numbers JQ715720 to JQ715775.

## Results

### Study Group

The age of the 289 patients with diarrhea ranged from 4 days to 55 months, with a mean age of 11 months (*S* = 7.8). The male to female gender ratio was 2.4 (204/85), and the difference in the age distributions of the boys and girls was not statistically significant (*P* = 0.7823).

### Epidemiology of Hospitalized HPeV Infections

Of the 289 specimens, 73 (25.3%, 73/289) were HPeV-positive. Among the positive specimens, 54 (74.0%, 54/73) were from boys and the rest from girls. However, taking into account the gender ratio, the prevalence of HPeV was gender neutral (26.5% versus 22.4%, *P* = 0.4629). The age distribution of these positive cases was from 2 to 35 months; 20.5% (15/73) were younger than 6 months, 52.0% (38/73) were 7 to 12 months old and 23.3% (17/73) were 13 to 24 months old.

**Figure 1:**
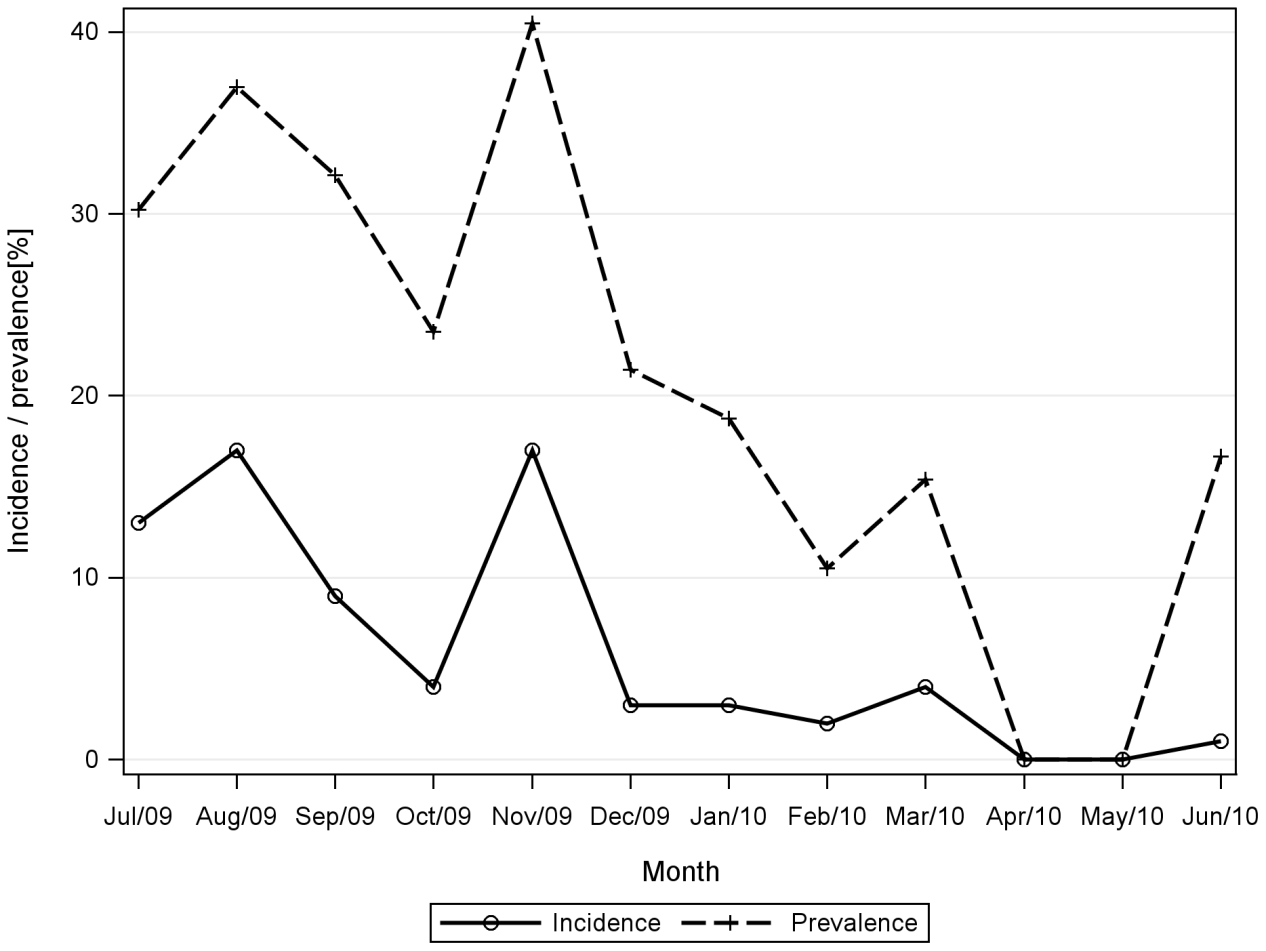
Seasonal distribution of HPeV-positive samples. Incidence means HPeV positive cases per month, prevalence means HPeV positive rate per month.

The monthly incidence (i.e. new HPeV positive cases per month) trend was consistent with the prevalence (HPeV positive rate per month) and showed an evident seasonality throughout the study period. HPeV was more prevalent during July–September and November, with two peak periods in “August and November” and a minimum in “April through May” (Figure 1).

### Phylogenetic Analysis

**Figure 2:**
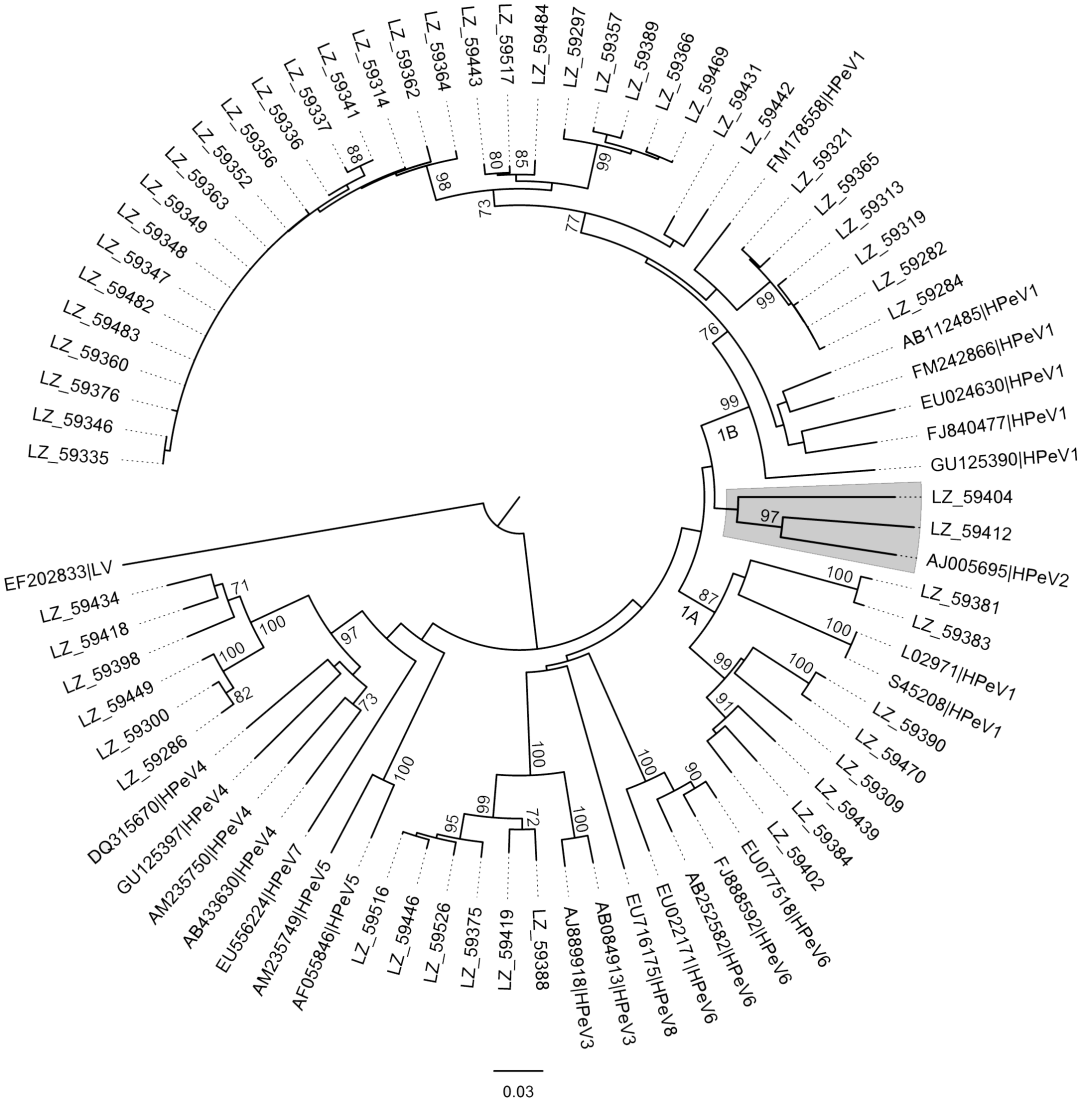
Nucleic acid phylogenetic tree based on the VP3/VP1 conjunction region. The phylogenetic analyses were conducted in MEGA5 by using the Neighbor-Joining method. The p-distance method was applied to compute the evolutionary distances. LV was used as outgroup. A bootstrap test was replicated 1000 times and only the bootstrap values >70 are displayed. The reference strains are shown with their individual GenBank accession number and corresponding genotype.

**Figure 3:**
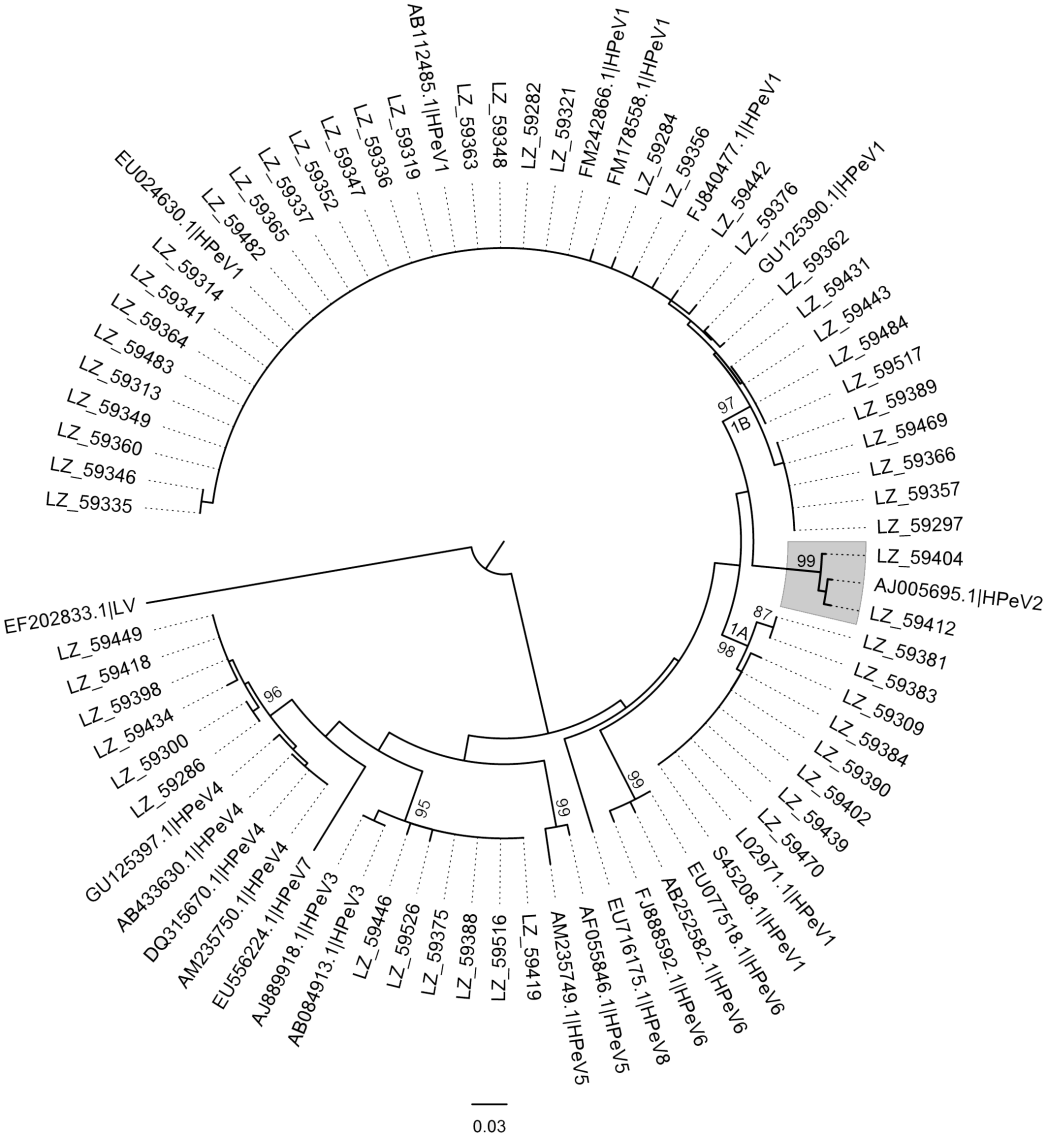
Amino acid phylogenetic tree based on the VP3/VP1 conjunction region. The phylogenetic analyses were conducted in MEGA5 by using the Neighbor-Joining method. The p-distance method was applied to compute the evolutionary distances. LV was used as outgroup. A bootstrap test was replicated 1000 times and only the bootstrap values >70 are displayed. The reference strains are shown with their individual GenBank accession number and corresponding genotype.

Of 73 positive samples that had been subjected to the VP3/VP1 genotyping PCR, 56 (76.7%, 56/73) were successfully amplified and sequenced. Genotypes were determined based on the phylogenetic relationship with the reference strains from GenBank. HPeV genotypes 1 to 4 were identified, and the number of individual genotypes were as follows: 42 HPeV1, 2 HPeV2, 6 HPeV3 and 6 HPeV4. HPeV1 was the predominant genotype, the majority of them clustered with the contemporary reference strains in the tree, while some clustered with the prototype Harris strain (L02971 & S45208) and formed a separate clade. In the phylogenetic tree using nucleotide sequences (Figure 2), strain “LZ 59404” could not be assigned to any of the known genotypes and formed a separate branch. The pairwise distances (0.202–0.295) between this strain and other reference strains was over the threshold of 18% divergence for nucleotides for the division of intra- and intertype categories [29]. Using the amino acid sequences (Figure 3) strain “LZ 59404” was characterized as HPeV type 2 (pairwise distance between this strain and AJ005695.1 was 0.010, lower than the 8% divergence threshold). The mean pairwise distances of the typed HPeV1, 2, 3 and 4 were 0.104, 0.207, 0.033 and 0.062 (nucleotides) and 0.033, 0.020, 0.007 and 0.009 (amino acids), respectively.

The alignment result from the deduced amino acid sequences by ClustalX indicated that all of the HPeV sequences used in the phylogenetic analysis contained the highly conserved Q/N (aa 59/60) which is believed to be the putative cleavage site of VP3/VP1 (data not shown) [51], [52]. Within the nucleotide sequences an unexpected trinucleotide (GAT, nt 241–243) insertion was found in all of the HPeV4 sequences but not in the other genotypes.

### Prevalence of HPeV Genotypes

**Figure 4:**
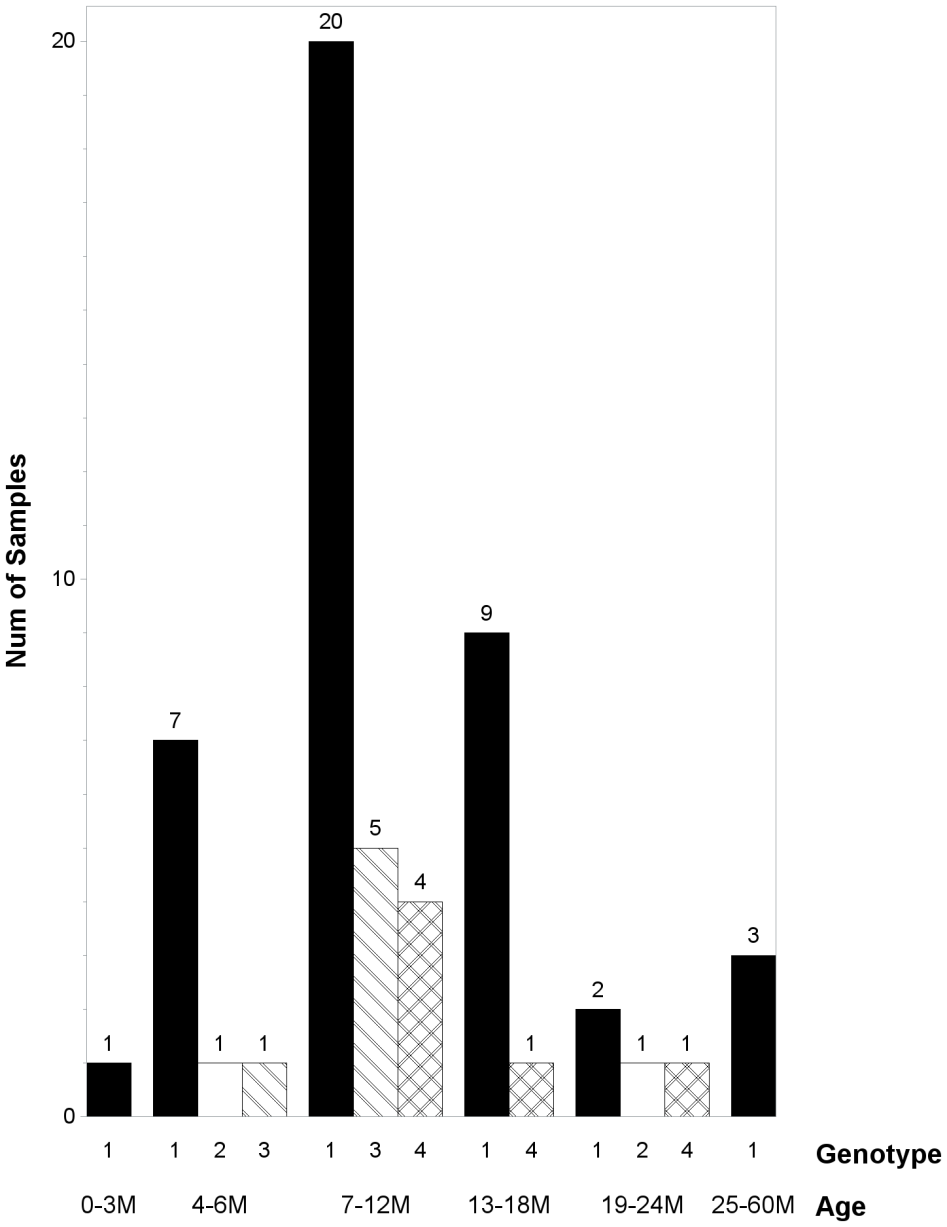
Age distribution of individual HPeV genotypes. Only the genotypes detected and successfully typed are indicated in each age group.

**Figure 5:**
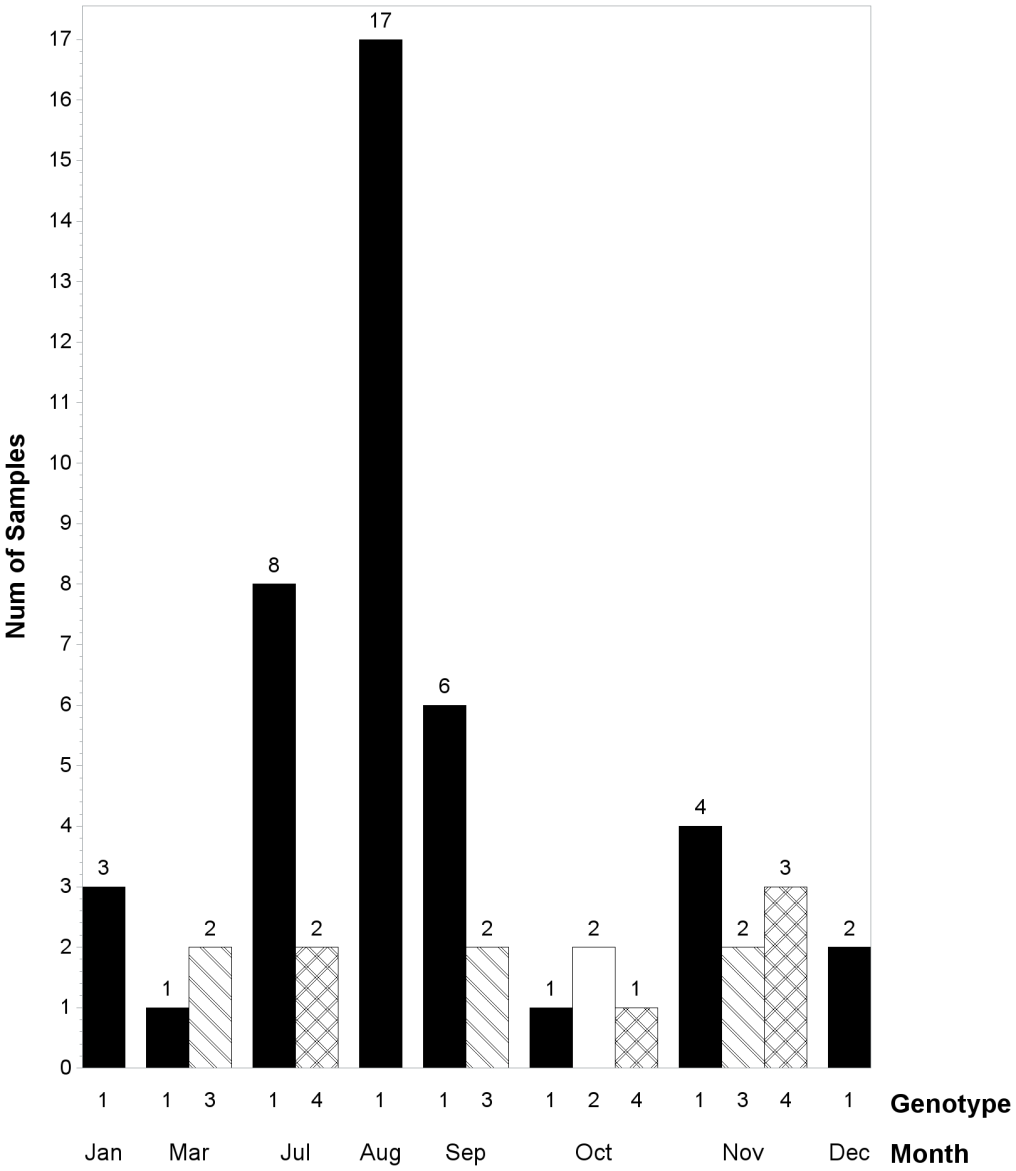
Seasonal distribution of individual HPeV genotype. February and April–June were not included because no genotypes had been successfully typed in these months. Only the genotypes detected and successfully typed are indicated in each age group.

HPeV1, the most prevalent genotype as indicated by the phylogenetic analysis, was found throughout all of the age groups (Figure 4). Most of the HPeV1 positive children (37/42, 88.1%) were younger than 18 months, with an average age of 12 months (*S* = 7.0) and a male-female ratio of 2.8 (31∶11, *P* = 0.6202). HPeV1 was primarily identified from specimens collected during July–September (31/42, 73.8%) with a peak in August, although a minor prevalence of HPeV1 was revealed during November–January. March and October showed a relatively low prevalence, and only one HPeV1 positive case was characterized in each of these two months. No HPeV1 cases were detected in February or April–June (Figure 5). Two HPeV2 positive specimens were all from boys (6 and 20 months old, respectively), both collected in October. HPeV3 was found in more boys than girls (5∶1) but with no statistically significant difference (*P* = 0.8106), with an average age of 9 months (*S* = 2.8) and an even distribution in March, September and November. Infections of HPeV4 were only seen in boys and were identified in July, October and November. The average age of the positive cases was 12 months (*S* = 5.1).

### Co-infection and Clinical Manifestation

**Figure 6:**
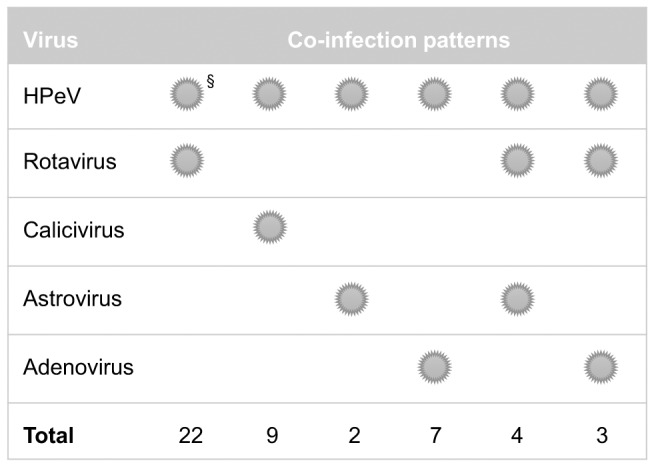
Co-infection of HPeV-infected subjects. § This symbol denotes the corresponding pathogen was involved in co-infection and the combination patterns of the symbol represented the modes of co-infections that had been identified in this study.

All of the fecal samples were also screened for rotavirus, astrovirus, calicivirus and adenovirus. Rotavirus was identified in 112 of the 289 samples, and 33 calicivirus-positive samples (22 positive for norovirus, 11 positive for sapovirus), 14 astrovirus-positive samples (13 positive for astrovirus 1, 1 positive for astrovirus 4), 31 adenovirus-positive samples (16 positive for adenovirus 41, 13 positive for adenovirus 40, 1 positive for adenovirus 12 and 1 positive for adenovirus 31) were also characterized in the 289 samples. The co-infection results are shown in Figure 6. Co-infections of HPeV with these four common diarrheal viruses accounted for 64.4% (47/73) of the HPeV-infected subjects, and 85.1% (40/47) of them were identified as dual infection. Rotavirus (29/47, 61.7%) was the most frequently co-detected pathogen. Among the sub-population not infected with any of the four common diarrheal viruses, HPeV showed a 22.2% (26/117) positive rate. Co-infection was seen in each of the identified genotypes HPeV1 to 4. No statistically significant difference was shown between the detection rates of rotavirus, astrovirus, calicivirus and adenovirus in HPeV-positive and negative children (Table 1).

**Table 1:** The detection rates of rotavirus, astrovirus, calicivirus and adenovirus in HPeV-infected and uninfected children.

Virus	Detection rate in HPeV-infected subjects	Detection rate in HPeV-uninfected subjects	*P* value
Rotavirus	39.7% (29/73)	38.4% (83/216)	0.8437
Calicivirus	12.3% (9/73)	11.1% (24/216)	0.7773
Astrovirus	8.2% (6/73)	3.7% (8/216)	0.2156
Adenovirus	13.7% (10/73)	9.7% (21/216)	0.3425

In addition to diarrhea, vomiting (42.3%, 11/26) and respiratory symptoms (11.5%, 3/26) were the other common symptoms in children with HPeV-infection alone. Clinical data of the HPeV-infected children with or without co-infection were compared. In addition, clinical data of HPeV-infected and non-infected subjects in the subgroup of children negative for any of the above four common diarrheal viruses were also compared (see Table 2). No statistically significant difference was seen in the statistical comparisons. One of the HPeV4 positive children without co-infection presented with accompanying abdominal pain. No fatal cases were documented.

**Table 2:** Comparison of clinical manifestations between children with HPeV-infection alone and HPeV-infected children with co-infection or children without pathogens detected.

Clinical data	HPeV-infected children with co-infection (A)	Children without pathogens detected (B)	Children with HPeV-infected alone (C)	*P* value†A *VS.* C	*P* value†B *VS.* C
Vomiting	53.2% (25/47)	42.8% (39/91)	42.3% (11/26)	0.3731	0.9602
Respiratory symptoms	23.4% (11/47)	18.7% (17/91)	11.5% (3/26)	0.3562	0.5769
Duration of diarrhea (days, mean± SD)	4±3.6	4±3.2	5±4.1	0.5389	0.7009
Duration of vomiting (days, mean± SD)	1±0.9	2±1.3	3±2.4	0.0325	0.1905
Frequency of diarrhea (times/day, mean± SD)	6±3.2	6±2.4	6±2.5	0.1603	0.4956
Frequency of vomiting (times/day, mean± SD)	4±2.6	3±2.3	4±1.8	0.6513	0.4759

† A Bonferroni-adjusted significance level of 0.025 is used for the multiple comparisons, and none of the comparisons are statistically significant.

## Discussion

The current annual prevalence of 25.3% for HPeV infection was comparable to the 29.4% detection rate that had been determined in the previous study conducted in 2006–2007 in the same region of China [30]. This implied a relatively constant prevalence rate of HPeV in this region. Again, in agreement with the previous study [30], nearly all the infections occurred in the first 2 years of life, and a larger proportion were between 7 to 12 months. The gender neutral distribution of HPeV infection also correlated with data from Thailand [53], Germany [33] and Shanghai, China [54].

Temporal variation in the seasonal distribution was revealed. In the present study, HPeVs were more frequently detected during July to November, which was different from the September to December preference observed in 2006–2007 [30]. The seasonal pattern of HPeV presented in the current study is more consistent with results from Shanghai, China which showed that the circulation of HPeV peaked in the summer and autumn, with high prevalence in July and August [54].

Changes in the circulation of HPeV genotypes were also revealed. More diverse genotypes of HPeV1, 3, 4, 6 and 8 were found circulating in 2006–2007 compared with the HPeV1, 2, 3 and 4 genotypes detected in 2009–2010. HPeV1 remained the predominant genotype, which was in common with previously reported data [10], [11], [14], [28], [30], [34], [53]–[58]; a similar age distribution but a difference in seasonality was shown, with the majority of the HPeV1 positive cases being clustered between July–September in 2009–2010 which varied from the October–December in 2006–2007 [30]. It is noteworthy that HPeV2, a rarely reported genotype, was detected unexpectedly in this study. As far as we know, this is the first report of HPeV2 prevalence in China to date. HPeV3, a commonly detected genotype globally and regarded second only to HPeV1 [10], [28], was detected in both periods but showed a clear distinction in the frequency (25 out of 286 in 2006–2007 compared to 6 out of 289 in 2009–2010, *P* = 0.004). The “biennial cycle” [6], [9]–[15], [32] which is a distinctive characteristic of this genotype may be an explanation for this fluctuation, but remains to be confirmed in future studies. A younger susceptible age to HPeV3 compared to HPeV1 was also revealed, which was contrary to the earlier study conducted in 2006–2007 [30]. Comparable positive rates of HPeV4 (2.0% in 2006–2007 compared to 1.4% in 2009–2010) displayed a low but relatively constant prevalence of this genotype in the study area. In 2006–2007, HPeV4 predominantly affected younger children, while in the present study it tended to be found in older children. Combining the data from these two studies, it can be concluded that HPeV4 displays a broad age distribution. HPeV6 and 8 which had been detected sporadically in 2006–2007 were not found in the present study. Low positive rates of HPeV6 and 8 were also found in the study from Shanghai [54]. No HPeV7 was identified all along, the absence of infection with this genotype was also noted in three other researches from China [54], [57], [59] indicating that this genotype is most likely absent in China. Therefore, combining the present and the previous data together [30], [54], [57], [59], the genotypes identified in China to date are seven (i.e. HPeV1–6 & 8).

As shown in most of the previous studies [10], [28], [33], [58], [60]–[64], HPeV1 constituted the largest group in the phylogenetic tree and formed two separate paraphyletic clades, namely HPeV1A and 1B. Most of the identified HPeV1 strains were clustered in the HPeV1B, only a few strains were found to be clustered with the prototype Harris strain which has been seldom found in recent years [30], [54]. This confirms that genotype HPeV1A is still circulating in China although to a lesser extent than HPeV1B. The inconsistent assignments in the nucleotide and amino acid trees of strain “LZ 59404” indicated that most of the differences in the nucleotide sequences between LZ 59404 and HPeV2 reference sequence AJ005695 occurred mainly in the synonymous sites. The relatively lower mean pairwise distance of the typed HPeV3 strains compared to the other genotype strains supported their recent origin and less genetic diversity [14], [65]. A Q/N conservation in the VP3/VP1 cleavage site was noted in all of the 79 sequences of HPeV1 to HPeV8 without exception, which corroborated the report from Watanabe *et al.* based on the sequences of HPeV1 to HPeV6 [51] and the preference for glutamine (Q) at the P1′ position [62], [66]. The unexpected and exclusive trinucleotide insertion of “GAT” into the HPeV4 nucleotide sequences still needs to be confirmed by sequencing more isolates to evaluate its influence on the biological behavior of this genotype.

The epidemiological profile of the co-infections was similar to the previous findings [30]. A 64.4% co-infection rate together with the previously reported 71.4% [30] and value of 66.7% from another report from Sri Lanka [34] suggest that co-infection is common for HPeV, and dual infection remained the main mode of co-infections. Rotavirus was still the most common co-infecting pathogen. When the samples that showed a positive result for any of the four viruses (e.g. rotavirus, astrovirus, calicivirus and adenovirus) were excluded, HPeV showed a 22.2% (26/117) detection rate in the present study. There have been three other studies which have screened stool samples negative for common enteric viruses for HPeV and have shown detection rates of 8.1% [58], 2.0% [55] and 14.6% [35] respectively. The discrepancy between values might be due to the different geographical locations and study populations. The non-significant difference between the detection rates of rotavirus, astrovirus, calicivirus and adenovirus in HPeV infected and uninfected groups suggests that there is no specific association between HPeV and any of the four common diarrheal viruses.

To date, most of studies have suggested a disease-causing role for HPeV, yet the former case-control study [30] and some other results [3], [28], [29] have reached contrasting conclusions. Recently, one report demonstrated that HPeV4 could be detected in fecal samples up to 40 days after the initial detection [67]. Two other longitudinal observations of Parechovirus in stool samples from Norwegian and Finnish infants revealed a 51-day median duration of an infection episode [28] and a maximum lasting shedding of 93 days [36], respectively. This long period of virus shedding in fecal samples may be an important consideration for studying the role of HPeV, especially when normal controls are included. While in the current study (without normal controls) the clinical profiles of the children with HPeV infected alone were not significantly different from the cases negative for HPeV and any of the four common diarrheal viruses, it once again suggested that HPeV might be not a causal factor for the pathogenesis of acute gastroenteritis or just as many other uncommon pathogens involved in diarrhea displaying a same degree of severity. Another factor that should be taken into account is that the severity of clinical manifestation of re-infection may be somewhat different from primary infection, which may influence the evaluation of the clinical relevance of HPeV to acute gastroenteritis. Due to a lack of serum samples from the study population, specific assays for the detection of IgM and IgG were not conducted in the present study. It would be informative to include such assays in future studies to determine the exact role of HPeV in the pathogenesis of acute gastroenteritis.

In summary, the prevalence, genetic diversity and clinical manifestations of HPeVs in children with acute gastroenteritis in Lanzhou, China were assessed and compared with previous findings [30]. Data revealed temporal variation in the seasonal distribution of HPeVs in this region and changing epidemiological patterns in HPeV genotypes over time. Continuous surveillance of HPeVs will provide a better understanding of the epidemiological changes of HPeVs over time and more information on new or uncommon genotypes of HPeV.
